# *trans*-Bis(1,3-diphenyl­propane-1,3-dionato)(methanol)oxidovanadium(IV) methanol disolvate

**DOI:** 10.1107/S1600536812044686

**Published:** 2012-10-03

**Authors:** Carla Pretorius, Johan A. Venter, Andreas Roodt

**Affiliations:** a Department of Chemistry, University of the Free State, PO Box 339, Bloemfontein 9300, South Africa

## Abstract

In the title compound, [V(C_15_H_11_O_2_)_2_O(CH_3_OH)]·2CH_3_OH, the V^IV^ atom is coordinated by two 1,3-diphenyl­propane-1,3-dionate ligands and an oxide ligand in an axial position. The sixth position is occupied by the O atom of a methanol group bonded *trans* to the oxide atom. The octa­hedral geometry is significantly distorted, with the V^IV^ atom lying 0.330 (3) Å above the equatorial plane formed by the O atoms of the two β-diketonate ligands. In the crystal, O—H⋯O hydrogen bonds between the coordinating methanol group in the complex and the two methanol solvent mol­ecules lead to the formation of polymeric chains along the *c*-axis direction. Weak C—H⋯O contacts are also observed.

## Related literature

For synthetic background, see: Schilde *et al.* (1995 ▶). For other methanol-substituted vanadium complexes, see: Gao *et al.* (1998 ▶); Chen *et al.* (2004 ▶); Tasiopoulos *et al.* (1999 ▶). For meth­oxy-substituted vanadium complexes, see: Bansse *et al.* (1992 ▶).
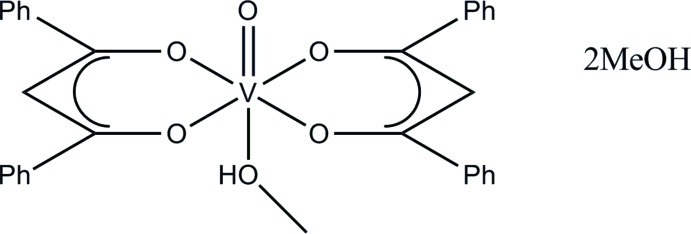


## Experimental

### 

#### Crystal data

[V(C_15_H_11_O_2_)_2_O(CH_4_O)]·2CH_4_O
*M* *_r_* = 609.54
Monoclinic, 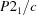

*a* = 16.1411 (1) Å
*b* = 10.7450 (6) Å
*c* = 18.5378 (13) Å
β = 113.579 (2)°
*V* = 2946.7 (3) Å^3^
*Z* = 4
Mo *K*α radiation
μ = 0.39 mm^−1^
*T* = 100 K
0.47 × 0.07 × 0.05 mm

#### Data collection

Bruker APEXII KappaCCD diffractometer
Absorption correction: multi-scan (*SADABS*; Bruker, 2008 ▶) *T*_min_ = 0.968, *T*_max_ = 0.981
38614 measured reflections
7317 independent reflections
5545 reflections with *I* > 2σ(*I*)
*R*_int_ = 0.046

#### Refinement

*R*[*F* ^2^ > 2σ(*F* ^2^)] = 0.041
*wR*(*F* ^2^) = 0.102
*S* = 1.03
7317 reflections
412 parameters
H atoms treated by a mixture of independent and constrained refinement
Δρ_max_ = 0.45 e Å^−3^
Δρ_min_ = −0.43 e Å^−3^

### 

Data collection: *APEX2* (Bruker, 2011 ▶); cell refinement: *SAINT-Plus* (Bruker, 2008 ▶); data reduction: *SAINT-Plus*; program(s) used to solve structure: *SHELXS97* (Sheldrick, 2008 ▶); program(s) used to refine structure: *SHELXL97* (Sheldrick, 2008 ▶); molecular graphics: *Mercury* (Macrae *et al.*, 2008 ▶); software used to prepare material for publication: *WinGX* (Farrugia, 1999 ▶), *publCIF* (Westrip, 2010 ▶), *PARST* (Nardelli, 1995 ▶) and *PLATON* (Spek, 2009 ▶).

## Supplementary Material

Crystal structure: contains datablock(s) global, I. DOI: 10.1107/S1600536812044686/sj5267sup1.cif

Structure factors: contains datablock(s) I. DOI: 10.1107/S1600536812044686/sj5267Isup2.hkl

Additional supplementary materials:  crystallographic information; 3D view; checkCIF report

## Figures and Tables

**Table 1:** Selected bond lengths (Å)

O1—V1	1.5965 (13)
O2—V1	1.9972 (12)
O3—V1	2.0045 (12)
O4—V1	1.9847 (12)
O5—V1	1.9935 (12)
O6—V1	2.3020 (15)

**Table 2:** Hydrogen-bond geometry (Å, °)

*D*—H⋯*A*	*D*—H	H⋯*A*	*D*⋯*A*	*D*—H⋯*A*
O6—H6*A*⋯O7	0.82 (3)	1.83 (3)	2.644 (2)	169 (3)
O7—H7*A*⋯O8^i^	0.87 (3)	1.90 (3)	2.749 (2)	168 (3)
O8—H8*A*⋯O3	0.90 (3)	1.96 (3)	2.853 (2)	178 (3)
C13—H13⋯O1^ii^	0.95	2.58	3.487 (2)	160
C32—H32*B*⋯O1^i^	0.98 (3)	2.43 (3)	3.360 (3)	159 (2)

Symmetry codes: (i) 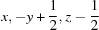; (ii) 
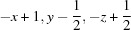
.

**Fig. 1.**
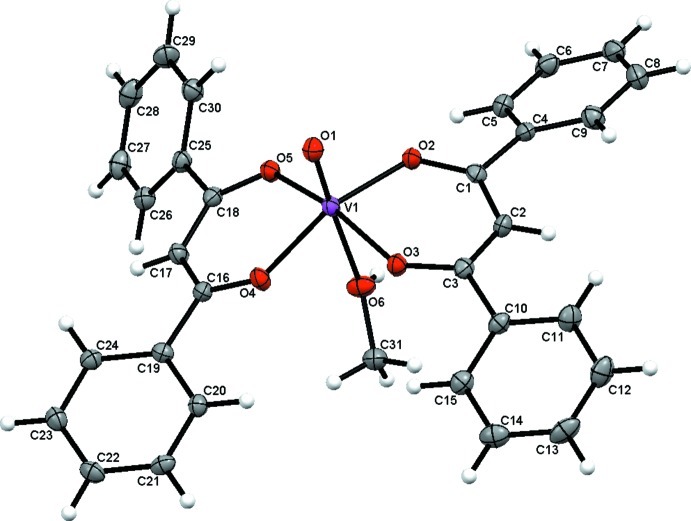
Molecular structure of the title compound. Displacement ellipsoids are drawn at the 50% probability displacement level. Solvent molecules have been omitted for clarity.

**Fig. 2.**
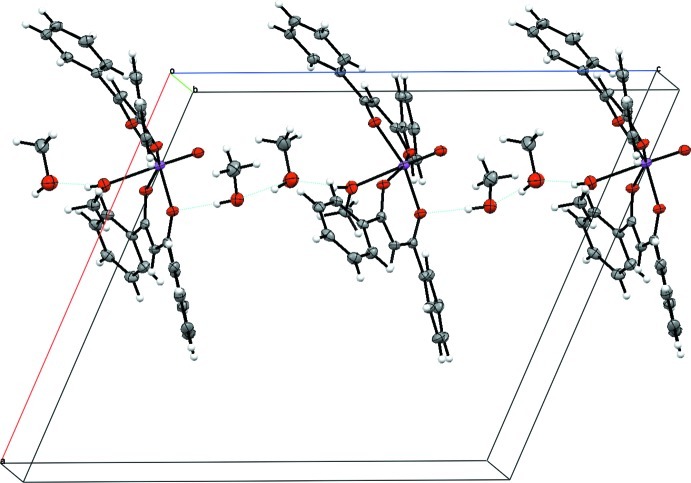
Hydrogen bonds (indicated in blue) linking one of the compound molecules and a solvent molecule leads to the formation of polymeric chains of the compound along the c-axis.

## supplementary crystallographic information

### Comment

The complex has two coordinated 1,3-diphenylpropane-1,3-dionato (dbm) ligands in the equatorial plane, the same as in the [VO(dbm)_2_] structure described by Schilde (Schilde *et al.*, 1995). The oxido group is in the axial position and no significant change in bond length is reported for the O1—V1 bond of 1.5964 (3) Å as compared to the [VO(dbm)_2_] structure (1.5922 (4) Å). The sixth coordination position at the vanadium centre *trans* to the oxido is occupied by a methanol molecule. The rather long bond length of 2.302 (2) Å is similar to methanol coordination in structures by Gao (2.346 Å) (Gao *et al.*, 1998), Chen (2.333 Å) (Chen *et al.*, 2004) and Tasiopoulos (2.301 Å) (Tasiopoulos *et al.*, 1999). A methoxy group bonded to a vanadium metal centre would have a V-OMe bond length of approximately 1.755 Å (Bansse *et al.*, 1992).

Intermolecular O6—H6A···O7 hydrogen bonding in the order of 2.644 (2) Å was observed with a methanol solvent molecule. Additional intermolecular hydrogen bonding was also noted between O7—H7A···O8^i^ of the order 2.749 (2) Å and O8—H8A···O3 in the order of 2.853 (2) Å. These interactions eventually lead to the formation of polymeric chains of the complex along the *c*-axis, as illustrated in Figure 2.

Weaker intermolecular hydrogen bonding was also noted between C13—H13···O1^ii^ in the order of 3.487 (2) Å and C32—H32*B*···O1^i^ in the order of 3.360 (3) Å.

### Experimental

V_2_O_5_ (1.0 g, 5.5 mmol) was added to a mixture of ethanol, water and sulfuric acid (5 cm^3^, 2 cm^3^ and 2 cm^3^ respectively) and refluxed for one hour, after which the yellow mixture turned a brilliant blue colour. A solution of 1,3-diphenylpropane-1,3-dione (4.93 g, 22 mmol) in ethanol (10 cm^3^) was added to the reaction mixture which was then stirred for *ca* 10 min. A saturated solution of sodium carbonate in water (20 cm^3^) was added to the mixture and the resulting green precipitate was collected by filtration. The precipitate was recrystallized from methanol and, after two weeks, small red needle-like crystals of [VO(dbm)_2_(MeOH)] were formed (yield: 2.35 g, 70%).

### Refinement

The methyl and aromatic H atoms were placed in geometrically idealized positions and constrained to ride on their parent atoms, with C—H = 0.95 and 0.98 Å and *U*_iso_(H) = 1.5Ueq(C) and 1.2Ueq(C), respectively. The hydrogen atoms of the methine groups, the methanol hydroxyl groups as well as the H atoms on C32 were located on the Fourier difference map and refined isotropically. The highest residual electron density was located 0.54 Å from H31C and the deepest hole was 0.68 Å from V1.

### Crystal data

**Table:**

[V(C_15_H_11_O_2_)_2_O(CH_4_O)]·2CH_4_O	*F*(000) = 1276
*M**_r_* = 609.54	*D*_x_ = 1.374 Mg m^−^^3^
Monoclinic, *P*2_1_/*c*	Mo *K*α radiation, λ = 0.71073 Å
Hall symbol: -P 2ybc	Cell parameters from 7652 reflections
*a* = 16.1411 (1) Å	θ = 2.2–27.7°
*b* = 10.7450 (6) Å	µ = 0.39 mm^−^^1^
*c* = 18.5378 (13) Å	*T* = 100 K
β = 113.579 (2)°	Needle, red
*V* = 2946.7 (3) Å^3^	0.47 × 0.07 × 0.05 mm
*Z* = 4	

### Data collection

**Table:**

Bruker APEXII KappaCCD diffractometer	7317 independent reflections
Radiation source: fine-focus sealed tube	5545 reflections with *I* > 2σ(*I*)
Graphite monochromator	*R*_int_ = 0.046
Detector resolution: 512 pixels mm^-1^	θ_max_ = 28.3°, θ_min_ = 2.2°
φ and ω scans	*h* = −21→21
Absorption correction: multi-scan (*SADABS*; Bruker, 2008)	*k* = −14→8
*T*_min_ = 0.968, *T*_max_ = 0.981	*l* = −24→24
38614 measured reflections	

### Refinement

**Table:**

Refinement on *F*^2^	Primary atom site location: structure-invariant direct methods
Least-squares matrix: full	Secondary atom site location: difference Fourier map
*R*[*F*^2^ > 2σ(*F*^2^)] = 0.041	Hydrogen site location: inferred from neighbouring sites
*wR*(*F*^2^) = 0.102	H atoms treated by a mixture of independent and constrained refinement
*S* = 1.03	*w* = 1/[σ^2^(*F*_o_^2^) + (0.0383*P*)^2^ + 2.0104*P*] where *P* = (*F*_o_^2^ + 2*F*_c_^2^)/3
7317 reflections	(Δ/σ)_max_ = 0.001
412 parameters	Δρ_max_ = 0.45 e Å^−^^3^
0 restraints	Δρ_min_ = −0.43 e Å^−^^3^
0 constraints	

### Special details

**Table:**

Geometry. All s.u.'s (except the s.u. in the dihedral angle between two l.s. planes) are estimated using the full covariance matrix. The cell s.u.'s are taken into account individually in the estimation of s.u.'s in distances, angles and torsion angles; correlations between s.u.'s in cell parameters are only used when they are defined by crystal symmetry. An approximate (isotropic) treatment of cell s.u.'s is used for estimating s.u.'s involving l.s. planes.
Refinement. Refinement of *F*^2^ against ALL reflections. The weighted *R*-factor *wR* and goodness of fit *S* are based on *F*^2^, conventional *R*-factors *R* are based on *F*, with *F* set to zero for negative *F*^2^. The threshold expression of *F*^2^ > 2σ(*F*^2^) is used only for calculating *R*-factors(gt) *etc*. and is not relevant to the choice of reflections for refinement. *R*-factors based on *F*^2^ are statistically about twice as large as those based on *F*, and *R*- factors based on ALL data will be even larger.

### Fractional atomic coordinates and isotropic or equivalent isotropic displacement parameters (Å^2^)

**Table:**

	*x*	*y*	*z*	*U*_iso_*/*U*_eq_	
C1	0.36765 (12)	0.39961 (16)	0.05770 (10)	0.0162 (4)	
C2	0.43706 (12)	0.32029 (17)	0.10546 (11)	0.0173 (4)	
C3	0.42559 (12)	0.20489 (16)	0.13327 (10)	0.0169 (4)	
C4	0.39167 (12)	0.52183 (16)	0.03351 (11)	0.0167 (4)	
C5	0.33514 (12)	0.57289 (17)	−0.03831 (11)	0.0200 (4)	
H5	0.2826	0.529	−0.0715	0.024*	
C6	0.35509 (13)	0.68762 (17)	−0.06167 (12)	0.0226 (4)	
H6	0.3166	0.7219	−0.111	0.027*	
C7	0.43106 (13)	0.75208 (17)	−0.01303 (12)	0.0234 (4)	
H7	0.4441	0.8312	−0.0288	0.028*	
C8	0.48820 (13)	0.70229 (17)	0.05840 (12)	0.0246 (4)	
H8	0.5405	0.7469	0.0913	0.03*	
C9	0.46897 (13)	0.58668 (17)	0.08203 (11)	0.0218 (4)	
H9	0.5082	0.552	0.1309	0.026*	
C10	0.50605 (12)	0.12914 (17)	0.18099 (10)	0.0177 (4)	
C11	0.58991 (13)	0.18430 (19)	0.22357 (11)	0.0226 (4)	
H11	0.5958	0.2723	0.2241	0.027*	
C12	0.66476 (13)	0.1110 (2)	0.26513 (12)	0.0274 (5)	
H12	0.7216	0.1492	0.2937	0.033*	
C13	0.65718 (14)	−0.0174 (2)	0.26531 (12)	0.0278 (5)	
H13	0.7086	−0.0672	0.2938	0.033*	
C14	0.57377 (14)	−0.07296 (19)	0.22355 (12)	0.0252 (4)	
H14	0.5682	−0.161	0.2236	0.03*	
C15	0.49862 (13)	−0.00043 (17)	0.18183 (11)	0.0205 (4)	
H15	0.4418	−0.0391	0.1537	0.025*	
C16	0.11816 (12)	−0.00936 (16)	−0.01969 (11)	0.0161 (4)	
C17	0.05536 (12)	0.05557 (16)	−0.08338 (11)	0.0179 (4)	
C18	0.05763 (12)	0.18349 (16)	−0.09553 (10)	0.0159 (4)	
C19	0.11005 (12)	−0.14649 (16)	−0.01373 (10)	0.0157 (4)	
C20	0.18785 (12)	−0.21569 (16)	0.02779 (10)	0.0172 (4)	
H20	0.2441	−0.1745	0.0539	0.021*	
C21	0.18344 (13)	−0.34458 (17)	0.03109 (11)	0.0191 (4)	
H21	0.2367	−0.3911	0.0592	0.023*	
C22	0.10189 (13)	−0.40532 (17)	−0.00637 (11)	0.0209 (4)	
H22	0.0993	−0.4936	−0.0049	0.025*	
C23	0.02358 (13)	−0.33711 (17)	−0.04628 (11)	0.0213 (4)	
H23	−0.0327	−0.3787	−0.0711	0.026*	
C24	0.02754 (12)	−0.20826 (16)	−0.04996 (11)	0.0178 (4)	
H24	−0.0261	−0.162	−0.0772	0.021*	
C25	−0.01309 (12)	0.24140 (16)	−0.16673 (11)	0.0173 (4)	
C26	−0.05067 (12)	0.17798 (17)	−0.23829 (11)	0.0200 (4)	
H26	−0.0338	0.0942	−0.2417	0.024*	
C27	−0.11255 (13)	0.23700 (19)	−0.30452 (11)	0.0237 (4)	
H27	−0.1369	0.1943	−0.3534	0.028*	
C28	−0.13888 (13)	0.35818 (19)	−0.29931 (12)	0.0253 (4)	
H28	−0.1816	0.3982	−0.3446	0.03*	
C29	−0.10314 (13)	0.42114 (18)	−0.22823 (12)	0.0255 (4)	
H29	−0.1222	0.5036	−0.2245	0.031*	
C30	−0.03947 (12)	0.36340 (17)	−0.16254 (11)	0.0211 (4)	
H30	−0.0137	0.4076	−0.1142	0.025*	
C31	0.33537 (13)	0.05156 (17)	−0.04292 (12)	0.0227 (4)	
H31A	0.3045	−0.0252	−0.0393	0.034*	
H31B	0.3928	0.0584	0.0031	0.034*	
H31C	0.3471	0.0497	−0.0909	0.034*	
C32	0.17157 (17)	0.2844 (3)	−0.21685 (16)	0.0405 (6)	
C33	0.24944 (16)	−0.0441 (2)	0.22012 (13)	0.0351 (5)	
H33A	0.2382	−0.078	0.2645	0.053*	
H33B	0.264	−0.1122	0.192	0.053*	
H33C	0.1953	−0.0006	0.1842	0.053*	
O1	0.19196 (8)	0.26454 (12)	0.11198 (8)	0.0212 (3)	
O2	0.28326 (8)	0.37464 (11)	0.03141 (7)	0.0186 (3)	
O3	0.34775 (8)	0.15408 (11)	0.11931 (7)	0.0182 (3)	
O4	0.18704 (8)	0.04098 (11)	0.03497 (7)	0.0188 (3)	
O5	0.11722 (8)	0.25860 (11)	−0.04987 (7)	0.0181 (3)	
O6	0.27966 (9)	0.15603 (13)	−0.04550 (8)	0.0239 (3)	
O7	0.26609 (11)	0.29178 (17)	−0.16870 (10)	0.0416 (4)	
O8	0.32366 (10)	0.04114 (15)	0.24890 (9)	0.0311 (3)	
V1	0.22608 (2)	0.21767 (3)	0.046818 (18)	0.01557 (9)	
H2	0.4932 (14)	0.3447 (18)	0.1177 (11)	0.017 (5)*	
H6A	0.2815 (17)	0.203 (2)	−0.0798 (15)	0.041 (7)*	
H7A	0.2918 (18)	0.340 (3)	−0.1908 (16)	0.052 (8)*	
H8A	0.3325 (19)	0.078 (3)	0.2091 (18)	0.065 (9)*	
H17	0.0095 (13)	0.0099 (18)	−0.1201 (12)	0.018 (5)*	
H32A	0.1461 (18)	0.210 (2)	−0.1987 (15)	0.049 (7)*	
H32B	0.1601 (19)	0.273 (3)	−0.2725 (19)	0.066 (9)*	
H32C	0.1388 (17)	0.365 (2)	−0.2115 (15)	0.045 (7)*	

### Atomic displacement parameters (Å^2^)

**Table:**

	*U*^11^	*U*^22^	*U*^33^	*U*^12^	*U*^13^	*U*^23^
C1	0.0170 (9)	0.0157 (8)	0.0157 (9)	−0.0014 (7)	0.0065 (7)	−0.0038 (7)
C2	0.0115 (9)	0.0199 (9)	0.0188 (9)	−0.0003 (7)	0.0044 (7)	−0.0004 (7)
C3	0.0156 (9)	0.0194 (9)	0.0144 (9)	0.0002 (7)	0.0049 (7)	−0.0023 (7)
C4	0.0149 (9)	0.0147 (8)	0.0223 (9)	0.0013 (7)	0.0091 (8)	−0.0006 (7)
C5	0.0154 (9)	0.0204 (9)	0.0238 (10)	−0.0005 (7)	0.0074 (8)	−0.0007 (8)
C6	0.0203 (10)	0.0221 (9)	0.0266 (10)	0.0050 (8)	0.0107 (8)	0.0062 (8)
C7	0.0243 (10)	0.0169 (9)	0.0342 (11)	0.0005 (8)	0.0173 (9)	0.0025 (8)
C8	0.0222 (10)	0.0211 (9)	0.0301 (11)	−0.0057 (8)	0.0100 (9)	−0.0044 (8)
C9	0.0216 (10)	0.0208 (9)	0.0203 (10)	−0.0014 (8)	0.0056 (8)	−0.0010 (7)
C10	0.0161 (9)	0.0209 (9)	0.0155 (9)	0.0028 (7)	0.0057 (7)	0.0029 (7)
C11	0.0199 (10)	0.0251 (9)	0.0197 (10)	0.0005 (8)	0.0046 (8)	0.0049 (8)
C12	0.0173 (10)	0.0373 (12)	0.0226 (10)	0.0020 (9)	0.0027 (8)	0.0070 (9)
C13	0.0238 (10)	0.0364 (11)	0.0225 (10)	0.0135 (9)	0.0086 (9)	0.0113 (9)
C14	0.0306 (11)	0.0234 (10)	0.0227 (10)	0.0067 (8)	0.0119 (9)	0.0064 (8)
C15	0.0201 (9)	0.0225 (9)	0.0172 (9)	0.0014 (8)	0.0057 (8)	0.0020 (7)
C16	0.0137 (8)	0.0160 (8)	0.0189 (9)	−0.0006 (7)	0.0070 (7)	−0.0018 (7)
C17	0.0162 (9)	0.0159 (8)	0.0178 (9)	−0.0017 (7)	0.0028 (8)	−0.0024 (7)
C18	0.0136 (8)	0.0189 (8)	0.0149 (9)	0.0012 (7)	0.0053 (7)	−0.0017 (7)
C19	0.0180 (9)	0.0145 (8)	0.0148 (9)	0.0004 (7)	0.0067 (7)	0.0000 (7)
C20	0.0156 (9)	0.0186 (8)	0.0160 (9)	−0.0013 (7)	0.0049 (7)	−0.0009 (7)
C21	0.0200 (9)	0.0187 (9)	0.0199 (9)	0.0055 (7)	0.0091 (8)	0.0037 (7)
C22	0.0270 (10)	0.0147 (8)	0.0224 (10)	−0.0009 (8)	0.0112 (8)	0.0015 (7)
C23	0.0208 (9)	0.0192 (9)	0.0238 (10)	−0.0042 (8)	0.0090 (8)	−0.0010 (8)
C24	0.0167 (9)	0.0182 (8)	0.0180 (9)	0.0016 (7)	0.0065 (7)	0.0006 (7)
C25	0.0142 (9)	0.0178 (8)	0.0176 (9)	−0.0004 (7)	0.0040 (7)	0.0016 (7)
C26	0.0178 (9)	0.0194 (9)	0.0213 (10)	−0.0010 (7)	0.0064 (8)	−0.0011 (7)
C27	0.0181 (10)	0.0332 (11)	0.0166 (9)	−0.0029 (8)	0.0036 (8)	0.0004 (8)
C28	0.0179 (9)	0.0318 (11)	0.0222 (10)	0.0022 (8)	0.0038 (8)	0.0095 (8)
C29	0.0223 (10)	0.0201 (9)	0.0296 (11)	0.0048 (8)	0.0058 (9)	0.0061 (8)
C30	0.0192 (9)	0.0196 (9)	0.0203 (10)	0.0002 (7)	0.0036 (8)	0.0001 (7)
C31	0.0214 (10)	0.0191 (9)	0.0273 (10)	0.0017 (8)	0.0095 (8)	−0.0016 (8)
C32	0.0373 (14)	0.0536 (16)	0.0319 (13)	−0.0119 (12)	0.0153 (11)	−0.0011 (12)
C33	0.0395 (13)	0.0309 (11)	0.0319 (12)	−0.0014 (10)	0.0111 (10)	0.0019 (9)
O1	0.0164 (6)	0.0228 (7)	0.0219 (7)	−0.0014 (5)	0.0050 (6)	−0.0022 (5)
O2	0.0136 (6)	0.0157 (6)	0.0228 (7)	−0.0008 (5)	0.0035 (5)	−0.0003 (5)
O3	0.0140 (6)	0.0173 (6)	0.0200 (7)	−0.0010 (5)	0.0034 (5)	0.0016 (5)
O4	0.0170 (6)	0.0152 (6)	0.0184 (7)	−0.0024 (5)	0.0009 (5)	0.0010 (5)
O5	0.0153 (6)	0.0155 (6)	0.0188 (7)	−0.0003 (5)	0.0019 (5)	−0.0004 (5)
O6	0.0289 (8)	0.0198 (7)	0.0249 (7)	0.0063 (6)	0.0129 (6)	0.0057 (6)
O7	0.0356 (9)	0.0487 (10)	0.0377 (10)	−0.0030 (8)	0.0115 (8)	0.0180 (8)
O8	0.0288 (8)	0.0391 (9)	0.0259 (8)	0.0001 (7)	0.0113 (7)	0.0019 (7)
V1	0.01259 (15)	0.01394 (14)	0.01701 (16)	−0.00089 (12)	0.00259 (12)	−0.00022 (12)

### Geometric parameters (Å, º)

**Table:**

C1—O2	1.278 (2)	C21—C22	1.382 (3)
C1—C2	1.404 (2)	C21—H21	0.95
C1—C4	1.488 (2)	C22—C23	1.391 (3)
C2—C3	1.383 (3)	C22—H22	0.95
C2—H2	0.88 (2)	C23—C24	1.389 (2)
C3—O3	1.298 (2)	C23—H23	0.95
C3—O3	1.298 (2)	C24—H24	0.95
C3—C10	1.487 (2)	C25—C30	1.390 (2)
C4—C5	1.391 (3)	C25—C26	1.396 (3)
C4—C9	1.398 (3)	C26—C27	1.388 (3)
C5—C6	1.387 (3)	C26—H26	0.95
C5—H5	0.95	C27—C28	1.385 (3)
C6—C7	1.383 (3)	C27—H27	0.95
C6—H6	0.95	C28—C29	1.385 (3)
C7—C8	1.383 (3)	C28—H28	0.95
C7—H7	0.95	C29—C30	1.387 (3)
C8—C9	1.393 (3)	C29—H29	0.95
C8—H8	0.95	C30—H30	0.95
C9—H9	0.95	C31—O6	1.427 (2)
C10—C11	1.397 (3)	C31—H31A	0.98
C10—C15	1.398 (2)	C31—H31B	0.98
C11—C12	1.389 (3)	C31—H31C	0.98
C11—H11	0.95	C32—O7	1.429 (3)
C12—C13	1.385 (3)	C32—O7	1.429 (3)
C12—H12	0.95	C32—H32A	1.02 (3)
C13—C14	1.391 (3)	C32—H32B	0.98 (3)
C13—H13	0.95	C32—H32C	1.04 (3)
C14—C15	1.388 (3)	C33—O8	1.431 (3)
C14—H14	0.95	C33—H33A	0.98
C15—H15	0.95	C33—H33B	0.98
C16—O4	1.286 (2)	C33—H33C	0.98
C16—C17	1.397 (2)	O1—V1	1.5965 (13)
C16—C19	1.487 (2)	O2—V1	1.9972 (12)
C17—C18	1.396 (2)	O3—V1	2.0045 (12)
C17—H17	0.92 (2)	O4—V1	1.9847 (12)
C18—O5	1.281 (2)	O5—V1	1.9935 (12)
C18—C25	1.492 (2)	O6—V1	2.3020 (15)
C19—C20	1.396 (2)	O6—H6A	0.82 (3)
C19—C24	1.396 (2)	O7—H7A	0.87 (3)
C20—C21	1.389 (2)	O8—H8A	0.90 (3)
C20—H20	0.95	V1—O3	2.0045 (12)
			
O2—C1—C2	124.94 (16)	C23—C24—H24	119.9
O2—C1—C4	115.89 (15)	C19—C24—H24	119.9
C2—C1—C4	119.16 (16)	C30—C25—C26	119.14 (17)
C3—C2—C1	125.94 (17)	C30—C25—C18	119.14 (16)
C3—C2—H2	116.6 (13)	C26—C25—C18	121.66 (16)
C1—C2—H2	117.4 (13)	C27—C26—C25	120.20 (17)
O3—C3—C2	124.48 (16)	C27—C26—H26	119.9
O3—C3—C2	124.48 (16)	C25—C26—H26	119.9
O3—C3—C10	115.71 (15)	C28—C27—C26	120.01 (18)
O3—C3—C10	115.71 (15)	C28—C27—H27	120
C2—C3—C10	119.79 (16)	C26—C27—H27	120
C5—C4—C9	119.63 (17)	C27—C28—C29	120.19 (18)
C5—C4—C1	119.09 (16)	C27—C28—H28	119.9
C9—C4—C1	121.27 (16)	C29—C28—H28	119.9
C6—C5—C4	120.29 (17)	C28—C29—C30	119.81 (18)
C6—C5—H5	119.9	C28—C29—H29	120.1
C4—C5—H5	119.9	C30—C29—H29	120.1
C7—C6—C5	119.86 (18)	C29—C30—C25	120.60 (18)
C7—C6—H6	120.1	C29—C30—H30	119.7
C5—C6—H6	120.1	C25—C30—H30	119.7
C6—C7—C8	120.56 (18)	O6—C31—H31A	109.5
C6—C7—H7	119.7	O6—C31—H31B	109.5
C8—C7—H7	119.7	H31A—C31—H31B	109.5
C7—C8—C9	119.92 (18)	O6—C31—H31C	109.5
C7—C8—H8	120	H31A—C31—H31C	109.5
C9—C8—H8	120	H31B—C31—H31C	109.5
C8—C9—C4	119.74 (18)	O7—C32—H32A	108.0 (15)
C8—C9—H9	120.1	O7—C32—H32A	108.0 (15)
C4—C9—H9	120.1	O7—C32—H32B	111.6 (17)
C11—C10—C15	118.96 (17)	O7—C32—H32B	111.6 (17)
C11—C10—C3	121.47 (16)	H32A—C32—H32B	109 (2)
C15—C10—C3	119.55 (16)	O7—C32—H32C	110.7 (14)
C12—C11—C10	120.26 (18)	O7—C32—H32C	110.7 (14)
C12—C11—H11	119.9	H32A—C32—H32C	110 (2)
C10—C11—H11	119.9	H32B—C32—H32C	108 (2)
C13—C12—C11	120.54 (19)	O8—C33—H33A	109.5
C13—C12—H12	119.7	O8—C33—H33B	109.5
C11—C12—H12	119.7	H33A—C33—H33B	109.5
C12—C13—C14	119.54 (18)	O8—C33—H33C	109.5
C12—C13—H13	120.2	H33A—C33—H33C	109.5
C14—C13—H13	120.2	H33B—C33—H33C	109.5
C15—C14—C13	120.31 (18)	C1—O2—V1	127.28 (11)
C15—C14—H14	119.8	C3—O3—V1	126.95 (11)
C13—C14—H14	119.8	C16—O4—V1	128.73 (11)
C14—C15—C10	120.38 (18)	C18—O5—V1	127.87 (11)
C14—C15—H15	119.8	C31—O6—V1	128.35 (12)
C10—C15—H15	119.8	C31—O6—H6A	107.1 (18)
O4—C16—C17	124.21 (16)	V1—O6—H6A	122.5 (18)
O4—C16—C19	115.61 (15)	C32—O7—H7A	109.3 (18)
C17—C16—C19	120.13 (16)	C33—O8—H8A	111.0 (19)
C18—C17—C16	124.84 (17)	O1—V1—O4	101.25 (6)
C18—C17—H17	118.1 (12)	O1—V1—O5	99.43 (6)
C16—C17—H17	117.1 (12)	O4—V1—O5	89.13 (5)
O5—C18—C17	125.10 (16)	O1—V1—O2	99.25 (6)
O5—C18—C25	115.35 (15)	O4—V1—O2	159.47 (6)
C17—C18—C25	119.55 (16)	O5—V1—O2	88.66 (5)
C20—C19—C24	119.11 (16)	O1—V1—O3	98.18 (6)
C20—C19—C16	118.82 (16)	O4—V1—O3	86.02 (5)
C24—C19—C16	122.05 (16)	O5—V1—O3	162.32 (5)
C21—C20—C19	120.30 (17)	O2—V1—O3	89.94 (5)
C21—C20—H20	119.8	O1—V1—O3	98.18 (6)
C19—C20—H20	119.8	O4—V1—O3	86.02 (5)
C22—C21—C20	120.26 (17)	O5—V1—O3	162.32 (5)
C22—C21—H21	119.9	O2—V1—O3	89.94 (5)
C20—C21—H21	119.9	O3—V1—O3	0.00 (12)
C21—C22—C23	119.92 (17)	O1—V1—O6	177.74 (6)
C21—C22—H22	120	O4—V1—O6	80.77 (5)
C23—C22—H22	120	O5—V1—O6	81.56 (5)
C24—C23—C22	120.10 (17)	O2—V1—O6	78.71 (5)
C24—C23—H23	120	O3—V1—O6	80.89 (5)
C22—C23—H23	120	O3—V1—O6	80.89 (5)
C23—C24—C19	120.27 (17)		

### Hydrogen-bond geometry (Å, º)

**Table:**

*D*—H···*A*	*D*—H	H···*A*	*D*···*A*	*D*—H···*A*
O6—H6*A*···O7	0.82 (3)	1.83 (3)	2.644 (2)	169 (3)
O7—H7*A*···O8^i^	0.87 (3)	1.90 (3)	2.749 (2)	168 (3)
O8—H8*A*···O3	0.90 (3)	1.96 (3)	2.853 (2)	178 (3)
C13—H13···O1^ii^	0.95	2.58	3.487 (2)	160
C32—H32*B*···O1^i^	0.98 (3)	2.43 (3)	3.360 (3)	159 (2)

Symmetry codes: (i) *x*, −*y*+1/2, *z*−1/2; (ii) −*x*+1, *y*−1/2, −*z*+1/2.
